# Critical innovations in the assembly of the modern flight apparatus in Early Cretaceous birds

**DOI:** 10.1016/j.isci.2026.115506

**Published:** 2026-03-26

**Authors:** Qian Wu, Thomas A. Stidham, Jingmai K. O’Connor, Alida M. Bailleul, Zhonghe Zhou, Zhiheng Li

**Affiliations:** 1 Key Laboratory of Vertebrate Evolution and Human Origins, Institute of Vertebrate Paleontology and Paleoanthropology, Chinese Academy of Sciences, Beijing 100044, China; 2 Department of Biology, Austin College, Sherman, TX 75090, USA; 3 Negaunee Integrative Research Center, Field Museum of Natural History, Chicago, IL 60605, USA; 4 University of Chinese Academy of Sciences, Beijing 100049, China

**Keywords:** Paleontology, Biological sciences, Evolutionary biology, Paleobiology

## Abstract

A pivotal innovation in the evolution of powered flight in dinosaurs was the triosseal canal—a specialized passage formed by the scapula, coracoid, and furcula that guides the wing-elevation tendon. However, the origins of this structure remained obscure. Here, we applied integrated histological analysis and micro-computed tomography (CT) scanning of a new enantiornithine specimen and the basal ornithuromorph *Archaeorhynchus*. Our results indicate that the triosseal canal evolved first through paedomorphosis of the coracoscapular joint into a synchondrosis in ornithothoracines and subsequent acquisition of the acrocoracoclavicular joint in ornithuromorphs. The complete lack of a connection between the furcula and coracoid represents one of the crucial skeletal disparities between enantiornithines and ornithuromorphs. We propose that the closure of the triosseal canal in ornithuromorphs markedly improved tendon stability, facilitating a greater range of wing motion and more efficient flight compared to enantiornithines, serving as one of the critical functional triggers responsible for their ecological diversification.

## Highlights

• Ornithothoracines possess a paedomorphic cartilaginous coracoscapular joint
• Enantiornithines lacked an acrocoracoclavicular joint and a closed triosseal canal
• Ornithuromorphs evolved a closed triosseal canal via the acrocoracoclavicular joint
• Canal closure enhanced flight performance and drove ornithuromorph diversification

## Graphical abstract

**Figure:**
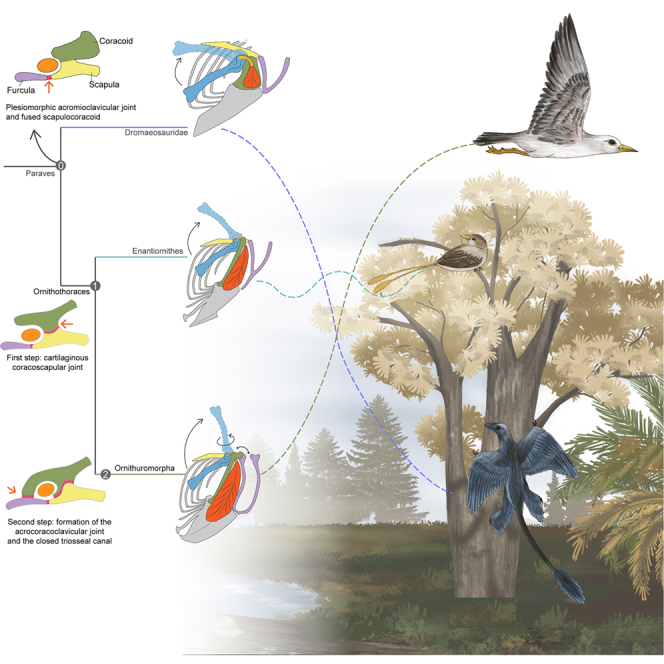

## Introduction

**Figure 1:**
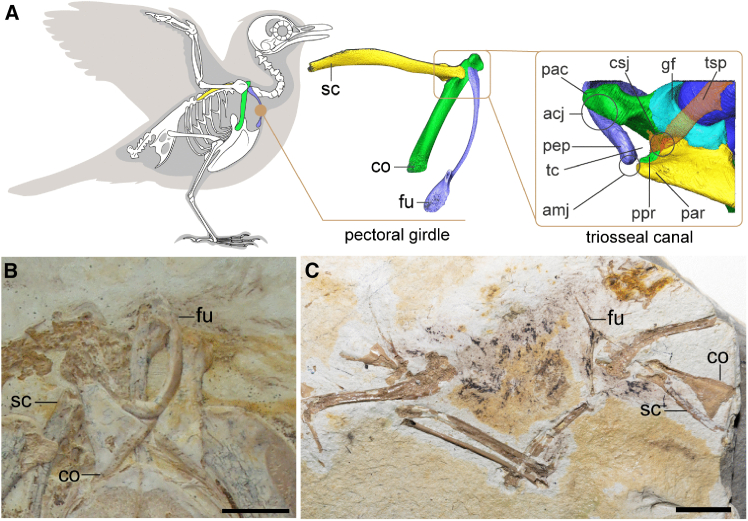
Pectoral girdle of crown birds and photographs of *Archaeorhynchus* (IVPP V14287) and Enantiornithes indet. (IVPP V12628) (A) pectoral girdle and triosseal canal of crown birds; (B) pectoral girdle of *Archaeorhynchus* IVPP V 14287; (C) Enantiornithes indet. IVPP V 12628. Abbreviations: acj, acrocoracoclavicular joint; amj, acromioclavicular joint; co, coracoid; csj, coracoscapular joint; fu, furcula; gf, glenoid fossa; pac, acrocoracoid process; par, acromion; pep, epicleidial process; ppr, procoracoid process; sc, scapula; tc, triosseal canal; tsp, tendon of *m. supracoracoideus*. Scale bars, 1 cm.

Birds represent the only living reptilian clade capable of powered flight and constitute the most species-rich group of aerial vertebrates.^1^^,^^2^ Their remarkable flight apparatus enables diverse aerial behaviors and results in a near-global distribution.^2^^,^^3^ Modern avian flight is powered by two major pectoral muscles originating primarily on the sternum^3^: the *m. pectoralis*, which pulls the wing down, generating thrust and lift, and the *m. supracoracoideus*, which elevates the wing via a unique pulley system.^4^^,^^5^ This pulley mechanism involves the tendon of *m. supracoracoideus* passing through the triosseal canal**—**a bony passage formed by the scapula, coracoid, and furcula**—**before attaching to the deltopectoral crest of the humerus (Figure 1A).^3^^,^^4^^,^^6^ During the avian flight stroke, contraction of the *m. supracoracoideus* pulls the deltopectoral crest dorsally and caudally,^4^^,^^5^^,^^7^ causing the globe-shaped humeral head to slide vertically and rotate (around the humeral long axis) within the concave articular surface (glenoid fossa) formed by the scapula and coracoid, rapidly elevating and rotating the wing.^4^^,^^5^^,^^7^^,^^8^^,^^9^^,^^10^ This rapid elevation and rotation of the wing are crucial for minimizing the duration of the upstroke to limit lift loss^11^ and maximize lift production during the downstroke.^4^ This integrated bone-ligament-tendon system stabilizes the wing and facilitates efficient flapping flight in modern birds. Morphological studies evaluating the pectoral girdle that relate to the evolution of flapping flight in dinosaurs have documented modifications in the orientation of the humeral glenoid fossa, elongation of the coracoid,^10^^,^^12^ and the appearance of the acrocoracoid process.^10^^,^^13^^,^^14^^,^^15^ These changes are inferred to have allowed greater dorsal excursion of the forelimb, shifting from the ancestral grasping motion, which was primarily cranially and laterally oriented, toward a flight-adapted stroke. However, comparatively little research has focused on the evolution of the triosseal canal. In *Archaeopteryx*, the most basal avian, the pectoral girdle exhibits a laterally oriented glenoid, a short coracoid, and no triosseal canal, suggesting that the earliest birds had very limited flight abilities.^15^^,^^16^

The term triosseal canal refers to the passage demarcated by three pectoral elements: formed by the acromion process of the scapula, the acrocoracoid process of the coracoid (synonyms: *extremitas omalis coracoidei*), and the epicleidial process (synonyms: *epicleidium*, acromial process, e*xtremitas omalis claviculae*, *extremitas scapularis*) of the furcula. Specifically, the canal is enclosed by three joints between these bones: the acromioclavicular joint between the scapula and the furcula; the coracoscapular joint between the coracoid and scapula; and the acrocoracoclavicular joint between the coracoid and furcula. Of these, only the acromioclavicular joint between the scapular acromion and furcula is plesiomorphically present in non-avian theropods.^14^^,^^17^ In contrast, the scapula and coracoid are typically fused in early theropods, with no intervening joint. The earliest appearance of the acrocoracoclavicular joint and transformation of the coracoscapular contact during early avian evolution remain unclear.

Joint structure determines the nature of bone articulation and the range of limb movement in the vertebrate skeleton.^18^^,^^19^^,^^20^ Thus, alongside other skeletal modifications, changes in joint structure within the pectoral girdle played a crucial role in the emergence of dinosaurian flight and the subsequent refinement of aerial locomotion, culminating in the sophisticated flight capabilities of modern birds.^2^ Investigating the evolutionary assembly of the joints that form the triosseal canal is therefore critical for understanding the early evolution of avian flight.

The acrocoracoclavicular joint, which contributes to the medial boundary of the triosseal canal, is absent in non-avian theropods and stemward (basal) birds (i.e., *Archaeopteryx* and basal pygostylian *Sapeornis*) because of the weak projection of the acrocoracoid process and the obtuse, boomerang-shaped furcula.^15^^,^^17^^,^^21^ In Ornithothoraces—the derived clade of Mesozoic birds consisting of Enantiornithes (the dominant bird group in the Cretaceous) and Ornithuromorpha (the clade that includes modern birds)^22^—the interclavicular angle is acute. However, because early bird fossils are typically preserved in two dimensions, the arrangement of the acrocoracoclavicular joint can be difficult to discern.^6^^,^^14^^,^^17^^,^^23^ A recent computed tomography (CT) scanning-based three-dimensional (3D) study of an enantiornithine shoulder girdle showed a significant gap between the acrocoracoid and epicleidial processe*s* in *Piscivorenantiornis*.^14^ Nevertheless, it was proposed that these two bony processes were connected by a ligament—as in some crown birds—thereby closing the medial margin of the triosseal canal.^14^^,^^24^ Adult non-avian theropods possess a fused scapulocoracoid.^15^^,^^25^ In contrast, ornithothoracines exhibit fully separate scapulae and coracoids. In early-branching ornithuromorphs, the coracoscapular joint has been described as a mobile ball-and-socket articulation, with a concave coracoid surface and a convex scapular surface.^3^^,^^22^^,^^23^^,^^26^^,^^27^ In enantiornithines, the scapular facet of the coracoid is slightly convex, and the coracoid facet of the scapula is slightly concave, forming a joint that is considered to be less mobile compared to the ball-and-socket joint of ornithuromorphs.^15^^,^^22^^,^^25^

Interpretations regarding the mobile coracoscapular joint and ligamentous acrocoracoclavicular connection have yet to be validated from a histological perspective.^3^^,^^14^^,^^22^^,^^23^^,^^24^^,^^26^ Ligaments attach to bone through entheses (insertion sites),^28^^,^^29^^,^^30^ which can be identified unambiguously through osteohistological thin-sectioning and provide direct evidence for the type of joint occurring between two skeletal elements. Furthermore, in vertebrates, the articular cartilage is connected to subchondral bone through a layer of calcified cartilage. Given the potential preservation of cartilage tissues,^31^^,^^32^ the occurrence of calcified cartilage in fossils is considered a good indicator for the presence of articular cartilage. Histological features of joints also provide clues regarding the amount of stress incurred during bone development and normal movement. In the case of the shoulder joint in volant birds, osteohistology can provide clues regarding the intensity of wing flapping.

In this study, we examine the structure of the triosseal canal through X-ray micro-CT scanning and histological analysis in both extant birds and early Cretaceous ornithothoracines—represented by a new specimen of enantiornithine (Enantiornithes indet.) and the basal ornithuromorph, *Archaeorhynchus spathula*, in order to examine the evolutionary history of discrete osteological characters contributing to the triosseal canal (Figures 1B and 1C). Our data reveal an immobile, cartilaginous coracoscapular joint in both enantiornithines and ornithuromorphs, and the absence of a ligamentous connection between the coracoid and furcula in enantiornithines. We further discuss the implications of these findings for the evolution of the shoulder joint and triosseal canal, and how these morphological changes influenced the functional mechanics of powered flight in birds. This research offers further insights into the evolution of the avian shoulder girdle and advances our understanding of the biomechanical foundations of modern avian flight.

## Results

### Coracoscapular joint (coracoid-scapular articulation)

#### Ornithuromorpha

**Figure 2:**
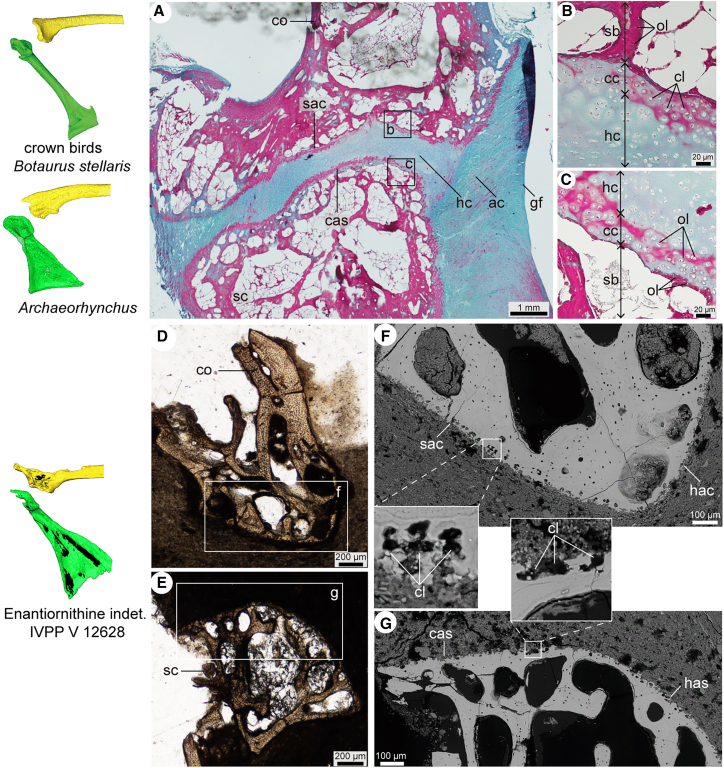
Morphology and Histology of the coracoscapular joint of fossil and extant birds Cross-section of the coracoscapular joint (A) and close-ups (B-C) of the coracoscapular joint of extant *B*. *stellaris*; cross-sections of the coracoid (D) and scapula (E) of enantiornithine IVPP V 12628, and close-up scanning electron microscope images (F, G). Abbreviations not listed in Figure 1 legend: ac, articular cartilage; cc, calcified cartilage; cl, chondrocyte lacuna; hc, hyaline cartilage; ol, osteocyte lacuna; and sb, subchondral bone.

In both the extant Eurasian bittern (*Botaurus stellaris*) and the basal ornithuromorph *A. spathula*, the scapular articular surface of the coracoid is concave, while the corresponding scapular surface is convex—a morphology typical of ornithuromorphs (Figure 2). Although this concave-convex arrangement was interpreted previously as evidence for a mobile ball-and-socket (synovial) joint,^3^^,^^22^^,^^23^^,^^26^ our histological data reveal that the two surfaces are connected by hyaline cartilage, forming an immobile synchondrosis (Figure 2A). This cartilaginous joint (formed by the scapula and coracoid) is also present in crown birds with a flat coracoscapular articular surface.^3^^,^^27^^,^^33^ Notably, the cartilage bridging the scapula and coracoid is continuous with the articular cartilage of the glenoid fossa, leaving no space for a synovial membrane or synovial cavity (Figures 2A–2C), thereby excluding the possibility of a synovial joint.

Therefore, the neornithine coracoscapular joint is unequivocally cartilaginous, as in the other extant archosaur lineage (e.g., crocodilians), regardless of the morphology of the articular surfaces.^34^ Based on the extant phylogenetic bracket of archosaurs and the conserved joint morphology between fossil and extant ornithuromorphs, we infer that Cretaceous ornithuromorphs also possessed a cartilaginous coracoscapular joint.

#### Enantiornithes

In the enantiornithine specimen IVPP V 12628, ground-section analysis of the glenoid fossa (i.e., humeral articular surfaces of the scapula and coracoid), the scapular articular surface of the coracoid, and the coracoid articular surface of the scapula revealed well-preserved calcified cartilage containing distinct chondrocyte lacunae under both light and scanning electron microscopy (Figures 2D–2F). This condition confirms that the scapular articular surface of the coracoid and the coracoid articular surface of the scapula were both covered by articular cartilage, which was continuous with the articular cartilage of the humeral glenoid fossa. This structural arrangement indicates that, as in ornithuromorphs (Figure 2A), the lateral aspect of the enantiornithine coracoscapular joint was formed by glenoid cartilage—not by a synovial membrane, which would be expected if it were a mobile synovial joint. Critically, unlike a pliable synovial membrane, cartilage resists twisting or extensive deformation without damage.^18^^,^^35^ If a synovial cavity had been present between the scapula and coracoid, movement would have ruptured the glenoid cartilage, comprising both the coracoscapular and the glenoid joints. Given these biomechanical constraints imposed by the glenoid cartilage and shared joint structure with ornithuromorphs, we conclude that the enantiornithine scapula and coracoid were connected via a synchondrosis.

### Acrocoracoclavicular joint (coracoid-furcular articulation)

#### Ornithuromorpha

**Figure 3:**
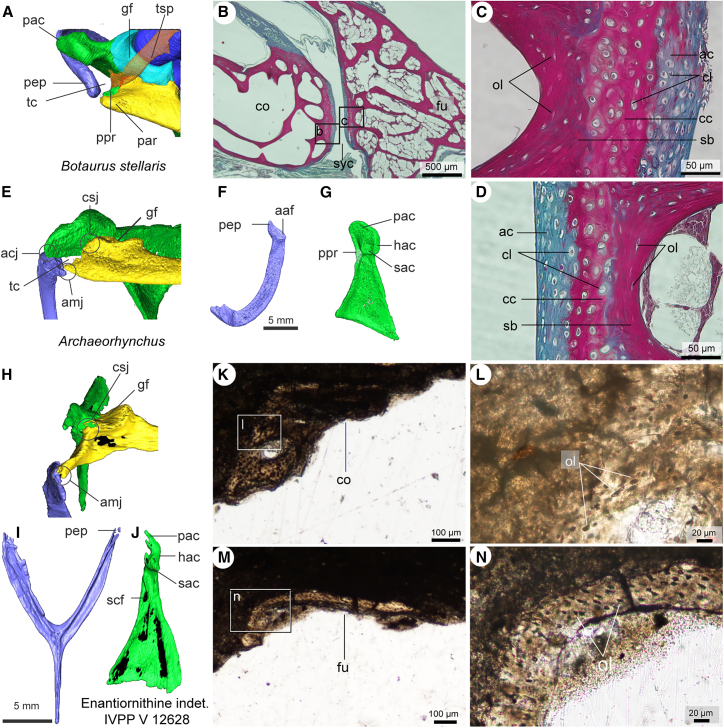
Pectoral girdle reconstruction and histology of fossil and extant bird specimens Pectoral girdle reconstruction in craniodorsal view of extant *Botaurus stellaris* (A); cross sections of the acrocoracoclavicular joint of extant *Apus apus* (B) and close-ups of the articular surface showing the articular cartilage (C-D), from Wu et al., 2021; pectoral girdle reconstruction in craniodorsal view (E), and furcula (F) and coracoid (G) in dorsal view of *Archaeorhynchus* IVPP V 14287, showing the presence of the acrocoracoclavicular joint, closed triosseal canal, and acrocoracoidal articular surface of furcula; pectoral girdle reconstruction in craniodorsal view (H), and furcula (I) and coracoid (J) in dorsal view; cross sections of the acrocoracoid process (K-L) and furcular omal end (M-N) of Enantiornithes indet. IVPP V 12628, showing the absence of the acrocoracoclavicular joint in enantiornithines. Abbreviations not listed in previous figures legends: syc, synovial cavity.

CT reconstruction of the articulated shoulder of *A. spathula* (IVPP V 14287) reveals that the dorsal surface of the epicleidial process contacts the omal end of the acromion process, while its lateral surface articulates with the medial side of the acrocoracoid process. These three elements form a fully enclosed triosseal canal, as observed in crown birds (Figures 1A and 3D).^3^ Given that *A. spathula* is among the most stem-ward known ornithuromorphs, this observation implies that the acrocoracoclavicular joint and a fully enclosed triosseal canal are synapomorphies of Ornithuromorpha (Figure S1).

#### Enantiornithes

The reconstructed shoulder girdle of IVPP V 12628 reveals that the dorsally oriented flat articular surface of the epicleidial process articulated with the flat cranial end of the scapular acromion, not with the coracoid (Figure 3G). This plesiomorphic feature is also present in non-avian theropods and stemward birds.^14^^,^^17^ A distinct gap separates the epicleidial process of the furcula from the acrocoracoid process of the coracoid, consistent with prior studies,^6^^,^^14^^,^^36^^,^^37^^,^^38^^,^^39^ confirming the absence of either a synostosis or a synovial joint between these two elements. Although a ligamentous connection (symphysis) has been proposed, analogous to some crown birds,^14^^,^^24^ thin sections of both processes (the acrocoracoid and the epicleidial processes) in IVPP V 12628 show no chondrocyte lacunae, fiber attachments, or other evidence of ligament enthesis. Only osteocyte lacunae are present within the bone matrix (Figures 3J–3M). This situation effectively rules out the presence of a closed triosseal canal.

Given the similar morphology between IVPP V 12628 and other enantiornithines with well-preserved pectoral girdles (e.g., *Concornis*, *Zhouornis*, *Parabohaiornis,* and *Yuanjiawaornis*) (Figures 3H and 3I),^14^^,^^40^^,^^41^^,^^42^^,^^43^^,^^44^^,^^45^^,^^46^ the absence of an acrocoracoclavicular joint and an enclosed triosseal canal likely represents the ancestral condition for Enantiornithes (Figure S1).

### Evolutionary history of triosseal canal structures

**Figure 4:**
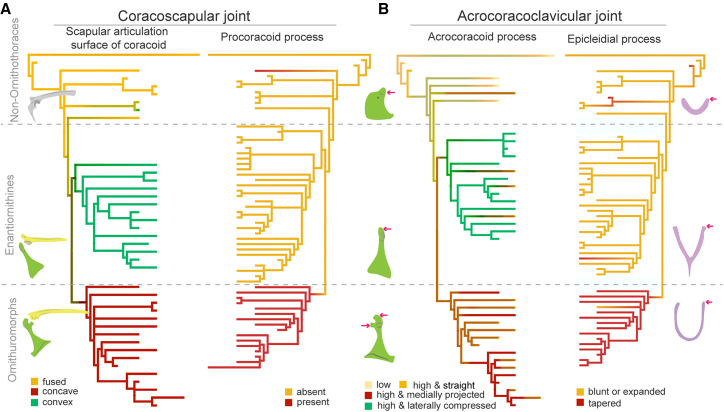
Early evolution of discrete morphological characters contributed to the triosseal canal (A) Ancestral state reconstruction of scapular articulation surface of the coracoid (left) and procoracoid process (right); (B) Ancestral state reconstruction of acrocoracoid process morphology (left) and furcular omal ends (right). See supplemental information for the species of each branch.

Ancestral state reconstruction reveals divergent trajectories of the coracoscapular joint evolution across basal avians, Enantiornithes, and Ornithuromorpha, while exhibiting remarkable homogeneity within individual clades (Figure 4A). Our results strongly support the emergence of a synchondrosis (previously described as “separated scapula and coracoid”) in the common ancestor of Ornithothoraces, with either a concave or convex scapular articular surface on the coracoid. Although unfused states have been reported in some basal birds, this pattern likely reflects ontogenetic variation that is widely documented in non-avian dinosaurs,^14^^,^^15^^,^^47^ and several specimens provide definitive evidence of fused elements in basal avian lineages (the Maxberg, Solnhofen, and Munich specimens of *Archaeopteryx*,^16^ Confuciusornithidae,^48^ *Jinguofortis* and *Chongmingia*^12^^,^^25^). Enantiornithines inherited the absence of the procoracoid process from their ancestral lineage, with independent acquisitions of procoracoid process in *Jeholornis*, *Protopteryx,* and ornithuromorphs. Given that the procoracoid process contributes to the coracoscapular joint complex, its presence shows limited diagnostic value for triosseal canal closure, consistent with observations in numerous extant birds that exhibit highly reduced procoracoid processes.^3^^,^^14^The coracoid-furcular articulation demonstrates greater morphological diversity and evolutionary complexity than the coracoscapular joint (Figure 4B). An acrocoracoid process positioned omal to the shoulder glenoid emerged in some basal avians and the ornithothoracine ancestor. A medially projected (or “hooked”) acrocoracoid process likely emerged in the ornithuromorph ancestor or through convergent evolution across multiple clades (e.g., *Archaeorhynchus*, *Mengciusornis*, and *Gansus*),^49^^,^^50^^,^^51^ facilitating the formation of the acrocoracoclavicular joint by reducing the acrocoracoid-furcular distance. Concurrently, the tapered omal ends of the furcula may help establish the acrocoracoclavicular joint in ornithuromorphs that have straight acrocoracoid processes, as in extant bird *B*. *stellaris* (Figure 2). Conversely, most enantiornithines retain plesiomorphic flat omal ends of the furcular and an autapomorphic small acrocoracoid process, morphologies consistent with the absence of their acrocoracoclavicular joints.

## Discussion

### Paedomorphic origin of the coracoscapular synchondrosis

**Figure 5:**
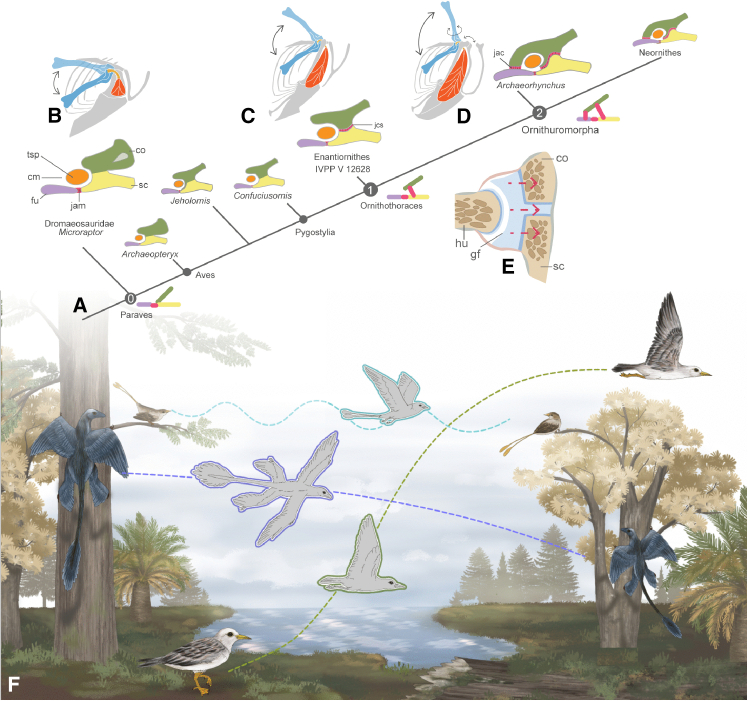
Hypothesized structure of the pectoral girdle joint, evolution of the triosseal canal, and flight style in Paraves (A) main steps of the formation of the triosseal canal are summarized: 0, the plesiomorphic acromioclavicular joint and fused scapulocoracoid in non-avian theropod and basal birds; 1, cartilaginous coracoscapular joint in ornithothoracine; 2, formation of the acrocoracoclavicular joint and the closed triosseal canal in Ornithuromorpha; hypothesized movement range of the humerus of Dromaeosauridae (B) based on *Microraptor*,^52^ Enantiornithes (C) based on IVPP V 12628 and Ornithuromorpha (D) based on Poore et al.^4^; (E) illustration of the general structure of the coracoscapular joint of ornithothoracines; showing the cartilaginous coracoscapular joint beneath the glenoid fossa in both enantiornithines and ornithuromorphs; (F) hypothesized flight style in Paraves. Phylogeny tree simplified from Chen et al. 2025. Abbreviations are the same as the previous figures' legends.

Histological analysis of the extant Eurasian bittern and the enantiornithine specimen IVPP V 12628 demonstrates that the ornithothoracine coracoscapular joint is an immobile synchondrosis, refuting prior hypotheses of a mobile synovial joint in ornithuromorphs (Figures 2 and 5E).^27^ This synchondrosis is conserved evolutionarily among archosaurs, as evidenced by its presence in extant crocodilians—the closest living relatives of birds.^34^ However, in pterosaurs and non-avian dinosaurs (including stem-group birds), this joint undergoes ontogenetic fusion, forming a rigid scapulocoracoid.^1^^,^^32^^,^^53^^,^^54^^,^^55^ The retention of a cartilaginous joint in ornithothoracines thus likely represents paedomorphosis—the persistence of an ancestrally juvenile trait into adulthood.^56^

While most temporary cartilaginous joints facilitate skeletal growth, the permanent synchondrosis in birds challenges previous hypotheses that linked shoulder girdle mobility to evolutionary changes in this joint.^18^^,^^57^ Although its precise function remains unclear, its cartilaginous nature suggests a role in stress absorption during forceful flapping.^18^ *In vivo* studies of pigeons and starlings reveal that their *m*. *supracoracoideus* can generate forces up to 7–10 times their body weight during flapping flight,^5^ transmitting mechanical stimuli through the humeral glenoid fossa to the coracoscapular joint (as depicted in Figure 5E). Such mechanical stimuli (including weight-bearing, movement, and tension), promote chondrogenesis and delay ossification,^18^^,^^33^^,^^58^ mirroring mechanisms observed in the human ischiopubic synchondrosis. In children, this pelvic joint enlarges asymmetrically because of mechanical loading from weight-bearing limbs.^59^^,^^60^ Conversely, reduced mechanical stimuli can lead to joint fusion, as observed in paralyzed chick embryos^61^ and rabbits.^62^ The fused scapulocoracoid in non-avian theropods and basal birds implies reduced contractile forces of the *m. pectoralis* and *m. supracoracoideus,* consistent with a less powerful flight stroke or grasping behavior in non-avian theropods. This interpretation is further supported by the absence of a large and cranially projecting sternal keel in non-ornithothoracine paravians, reflecting smaller flight muscle attachment areas compared to the enlarged sternal keel of ornithuromorphs.

### Completion of the triosseal canal: The acrocoracoclavicular joint in ornithuromorpha

The acrocoracoclavicular joint represents a key evolutionary innovation in ornithuromorphs, distinguishing them from other more stem-ward avian lineages and likely enhancing their powered flight capabilities (Figures 3, 4, and 5). In crown birds, the furcula undergoes mediolateral flexion (elastic deformation) during the flight stroke, which may improve flapping efficiency and/or assist in respiration.^9^^,^^17^^,^^63^^,^^64^^,^^65^ This flexion results from forces transmitted to the furcula via the acrocoracoclavicular joint, driven by movement of the coracoid’s sternal end within the sternal sulcus (*sulcus articularis coracoideus*).^9^ In contrast, enantiornithines and basal birds lack this joint, precluding coordinated coracoid-furcula movement and furcular flexion during flight. Interestingly, despite sharing narrow furcular rami with ornithuromorphs, enantiornithines must have relied on alternative mechanisms for force transmission—an intriguing question warranting further investigation.

The acrocoracoclavicular joint not only facilitates furcular flexion but also completes the triosseal canal, a hallmark of the neornithine flight apparatus.^3^^,^^21^ In early ornithuromorphs, the saddle-shaped glenoid joint articulates with a globular humeral head, while the tendon of the *m*. *supracoracoideus* passes through the enclosed triosseal canal to attach to the dorsal deltopectoral crest, which bears a distinct tubercle—as in crown birds (Figure 5D).^3^^,^^4^^,^^6^ Although a pulley-like tendon system exists in some non-ornithuromorph birds (*Jeholornis* and enantiornithines) (Figure 5C), their canal remains partially open (Figures 3G and 5A). We hypothesize that the enclosed triosseal canal in ornithuromorphs stabilized the tendon during the contraction of *m. supracoracoideus*, ensuring precise control of the humeral elevation and rotation—critical for expansive wing motion (elevation-depression, protraction, and rotation).

By contrast, enantiornithines exhibit a partially convex humeral head (convex caudally), with a concave cranial surface,^22^^,^^41^^,^^66^ which restricts rotation within the glenoid fossa (around the humeral long axis) (Figure 5C).^8^^,^^25^ This morphology likely prolonged the upstroke, increasing negative lift and reducing flight efficiency. Moreover, their open bony channel (formed by the acrocoracoid and scapular acromion) offered less stability for the tendon (Figure 5C). While still functional as a pulley,^14^ the tendon’s susceptibility to slippage would have further limited the humeral range of motion compared to that of crown birds. Collectively, the absence of a globular humeral head, acrocoracoclavicular joint, and enclosed triosseal canal suggests restricted flapping performance in enantiornithines—consistent with their smaller flight muscles, inferred from shallow and caudally restricted sternal keels.^3^^,^^67^^,^^68^^,^^69^^,^^70^

These anatomical differences align with reconstructions of enantiornithines as intermittent fliers (e.g., employing bounding or flapping-gliding flight) based on wing and forelimb morphology (e.g., *Concornis*, *Eoalulavis*, *Protopteryx*, and *Junornis*).^38^^,^^71^^,^^72^^,^^73^^,^^74^ Even *Bohaiornis*, though morphologically suited for continuous flight, was likely incapable of sustained flight,^75^ a conclusion supported by our research. Ground take-off, the most energetically demanding flight maneuver,^76^^,^^77^ would have been particularly challenging for enantiornithines, possibly requiring a running start to launch from the ground as inferred for *Archaeopteryx*.^78^

The restricted flight capability of enantiornithines (particularly their inability to perform continuous flight or efficiently take off from the ground) may have largely confined them to arboreal niches during the Early Cretaceous, as evidenced by their hindlimb and pedal claw morphology.^22^^,^^41^^,^^79^ In striking contrast, ornithuromorphs developed superior flapping capacity early in their evolution through morphological innovations such as the acrocoracoclavicular joint^23^^,^^80^ enabling ground (or water surface) takeoff comparable to that of crown birds. Their enhanced flight performance may have served as a key evolutionary breakthrough—a “functional trigger”—allowing ornithuromorphs to radiate into diverse terrestrial and aquatic habitats, a pattern reflected in the ecological variety of Cretaceous taxa.^81^ Notably, Late Cretaceous enantiornithines show significant increases in body size^82^^,^^83^^,^^84^ and adaptations such as the well-developed, cranially projecting keel in *Neuquenornis*,^85^ suggesting they eventually expanded their ecological and functional range. The absence of obvious osteological modifications in these later forms raises the possibility that some evolved a ligamentous (rather than osseous) closure of the triosseal canal—a hypothesis that could be tested through future histological analysis. Through integrated CT scanning and histological analysis, we have elucidated the evolutionary assembly of the triosseal canal—a fundamental component of the avian flight apparatus. Our results demonstrate that ornithothoracines evolved an immobile, cartilaginous coracoscapular joint via paedomorphosis, retaining the unfused juvenile condition of non-avian theropods into adulthood. This joint likely emerged in response to increased mechanical demands from more powerful wing flapping in derived avian lineages. This discovery challenges the long-standing assumptions of a ball-and-socket synovial joint between the scapula and coracoid in Mesozoic ornithuromorphs. Osteohistology confirms the complete absence of an acrocoracoclavicular joint in enantiornithines—not only bony but also ligamentous—indicating early enantiornithines lacked a fully enclosed triosseal canal. In contrast, this key innovation appears to have only evolved in ornithuromorphs, where it completed the modern flight apparatus and established a critical functional distinction between the two major Mesozoic avian lineages. Based on these results, we propose that during the evolution from non-avian paravians to crown birds, the triosseal canal evolved through two key steps of innovation: the cartilaginous coracoscapular joint in ornithothoracines and the acrocoracoclavicular joint in ornithuromorphs. The latter enabled furcular forces to be stored and transmitted during flight cycles. Combined with discrete differences in humeral head morphology—globular in ornithuromorphs versus caudally convex and cranially concave in enantiornithines—these skeletal features indicate that ornithuromorphs achieved a greater range of humeral motion and a faster, more coordinated wing upstroke. This morphology is consistent with predictions regarding intermittent flight styles in enantiornithines and potentially explains why enantiornithines were largely restricted to arboreal ecologies during the Early Cretaceous. This study advances our understanding of avian flight evolution by demonstrating how successive morphological innovations progressively refined flight capabilities during the Cretaceous radiation of birds.

### Limitations of the study

Despite providing histological insight into the pectoral girdle joints of early Cretaceous birds, this study is subject to limitations inherent to palaeobiological research. The scarcity of fossil specimens suitable for histological sampling precluded a high-resolution tracking of the morphological and histological evolution of these joints. Furthermore, while the extant phylogenetic bracket supports our inference of articular cartilage and ligaments, the absence of preserved soft tissue necessitates that reconstructions of joint mobility and loading regimes rely on indirect evidence. Consequently, the precise functional consequences of these joint configurations on flight kinematics remain to be quantified. A critical gap also remains regarding the late Cretaceous enantiornithines; specifically, the hypothesis that they independently evolved a ligamentous closure of the triosseal canal remains to be tested histologically. To resolve these uncertainties, future work must integrate broader taxonomic and ontogenetic sampling with a three-dimensional kinematic modeling approach to rigorously test these evolutionary and functional hypotheses.

## Resource availability

### Lead contact

Further information and requests should be directed to and will be fulfilled by the lead contact, Dr. Zhiheng Li (lizhiheng@ivpp.ac.cn).

### Materials availability

All specimens used in this study are deposited in the collection of IVPP in Beijing.

### Data and code availability

• All original data and code are available in this paper’s electronic supplemental information.
• Any additional information required to reanalyze the data reported in this paper is available from the lead contact upon request.

## Acknowledgments

We thank Yang J. M. for specimen preparation, Yin P. F. for help with CT scanning, Gao W. for photography, Wu Z. for illustration, and Wang S. Y. for discussion. We thank the High-Resolution X-ray Computed Tomography Laboratory and Microscopy Laboratory, Institute of Vertebrate Paleontology and Paleoanthropology, the 10.13039/501100002367Chinese Academy of Sciences, for providing the laboratory facilities and support. This research was supported by the 10.13039/501100001809National Natural Science Foundation of China (grant nos. 42302012 and 42288201) and the National Key Research and Development Project of China (2024YFF0807603).

## Author contributions

Q. W., Z. Z., A. B., J. O., T. S., and Z. L. designed the project. Q. W. and Z. L. collected and analyzed the data. Q. W., Z. Z., A. B., J. O., T. S., and Z. L. wrote the paper.

## Declaration of interests

The authors declare no competing interests.

## STAR★Methods

### Key resources table

**Table:** 

REAGENT or RESOURCE	SOURCE	IDENTIFIER
**Biological samples**		

Holotype specimen of *Archaeorhynchus spathula*	Institute of Vertebrate Paleontologyand Paleoanthropology (IVPP)	IVPP V 14287
Enantiornithine indet.	IVPP	IVPP V 12628
Skeleton of *Botaurus stellaris*	IVPP	OV2080

**Deposited data**		

Raw data, code for analyses herein, and additional results and discussion of extant data	This paper	

**Software and algorithms**		

Mesquite	Mesquite project	3.61
R programming language	R Core Team	4.4.3
Avizo	ThermoFisher Scientific Inc.	9.0

### Method details

#### Specimens

***Archaeorhynchus spathula****,* the phylogenetically most basal ornithuromorph discovered to date, represents the most primitive morphology of this clade.^86^^,^^87^ The holotype of *Archaeorhynchus* (IVPP V 14287) was scanned and digitally reconstructed (Figure 1B).

Enantiornithine indet. IVPP V 12628 is a disarticulated partial skeleton collected from the Lower Cretaceous Jiufotang Formation at Jinzhou City, Liaoning Province of China.^88^ The specimen, consisting of the forelimbs, a shoulder girdle, sternum, and some feather impressions (Figure 1C) is identified as an enantiornithine based on the presence of synapomorphies, including the concave proximal margin of the humerus, the ‘Y’-shaped furcula, the absence of the procoracoid process, and the slightly convex lateral margin of the coracoid. Its pectoral girdle exhibits typical Early Cretaceous enantiornithine morphology: craniodorsally deflected acromion process of the scapula, concave coracoidal articular surface on the scapula and convex scapular articular surface on the coracoid, craniocaudally expanded epicleidium, strut-like coracoid with slender and straight omal ends and lack of procoracoid process. Thus, it was chosen to represent the clade herein.

##### Extant specimen

The shoulder girdle of a great bittern (*Botaurus stellaris*) skeleton was used for triosseal canal reconstruction and coracoscapular joint histology analysis, to evaluate its “ball and socket” morphology in the joint (Figure 2). This specimen was donated to Institute of Vertebrate Paleontology and Paleoanthropology, Chinese Academy of Sciences (IVPP) by the Beijing Wildlife Rescue Center. All specimens used in this study are deposited in the extant collection of IVPP in Beijing. Phylogeny of fossil and extant species is simplified from a previous analysis (Figure 5).^25^

#### CT scanning and reconstruction

Both fossil and extant specimens were scanned using a 225 kV micro-CT scanner housed in the High-Resolution X-ray Computed Tomography Laboratory, Institute of Vertebrate Paleontology and Paleoanthropology (IVPP) of the Chinese Academy of Sciences in Beijing. Three-dimensional (3D) segmentation of the CT data was performed using the software Avizo 9.0, after CT scanning.

#### Ground sectioning and SEM (scanning electron microscopy) imaging of fossil samples

The shoulder end of the pectoral girdle of IVPP V 12628 was sectioned for histological analysis (see supplemental information for detailed procedures) in the Microscopy Laboratory of IVPP. The sectioned slices of the fossil specimens were analyzed at the Chinese Academy of Geological Sciences (Beijing) using FEI Quanta 450 (FEG) at 20 kV, employing BSE (back-scattered electrons) mode.

#### Paraffin sectioning and histochemistry staining of extant samples

The shoulder end of the extant specimen (*Botaurus stellaris*) was dissected, fixed and demineralized, then processed following a standardized protocol for paraffin sections.^89^ The sections then underwent a modified Masson’s trichrome (MT) stain.^90^ The paraffin section and stain of the extant specimen were performed in the Microscopy Laboratory of IVPP. See supplemental information for detailed procedures.

#### Ancestor state reconstruction

To determine the evolutionary history of the acrocoracoclavicular joint within Aves, we traced changes of this joint across a recently published phylogenetic framework of early birds using parsimony ancestral state reconstruction in Mesquite (v.3.61)^91^(Table S1). To further elucidate the key evolutionary steps of the triosseal canal in early birds, we formulated four discrete characters contributing to the coracoscapular and acrocoracoclavicular joints (morphology of the scapular articulation surface of coracoid, procoracoid process, acrocoracoid process and epicleidial process) among Jurassic and Early Cretaceous avians based on published works of characters and their states under the same phylogenetic framework (Table S2).^15^^,^^91^ Ancestral state reconstructions for discrete osteological characters were performed subsequently using stochastic character mapping analysis on a time-calibrated phylogeny with the “make.simmap” function in the R package *phytools*.^92^ See electronic supplemental information for the ‘R’ code and data.

#### Terminology

Terminology of osteology follows Baumel et al. (1993). Under this framework, the “clavicle” mentioned in certain terms is not an indicator of the homology of the furcula but part of the term. The term “bird” (sensu “Aves”) comprises the most recent common ancestor of *Archaeopteryx* and extant (crown-group) birds and all of its descendants,^93^ and “Ornithuromorpha” refers to the clade including all living birds and their closest relatives (with Enantiornithes excluded).^94^^,^^95^ The term Paraves is a stem-based taxon containing *Passer domesticus* and all coelurosaurians closer to it than to *Oviraptor philoceratops*.

### Quantification and statistical analysis

Our quantification and statistical analysis of the data included an ancestral states reconstruction approach that is explained in the method details.

## Supplemental information

Document S1. Figures S1–S5 and Methods S1 and S2

Table S1. Coracoid-furcular joint type of early birds

Table S2. State of characters contributing to the coracoscapular and acrocoracoclavicular joints among Jurassic and Early Cretaceous birds

Data S1. ‘R’ code and data used for ancestor state reconstruction
