# Supplementary material for: Critical innovations in the assembly of the modern flight apparatus in Early Cretaceous birds
Source: iScience. 2026 Mar 26;29(4):115506. doi: 10.1016/j.isci.2026.115506 (PMC13091450; doi:10.1016/j.isci.2026.115506)
Supplement: Document S1. Figures S1–S5 and Methods S1 and S2 [file mmc1.pdf]

iScience, Volume *29*

Supplemental information

Critical innovations in the assembly of the modern flight apparatus in Early Cretaceous birds

Qian Wu, Thomas A. Stidham, Jingmai K. O'Connor, Alida M. Bailleul, Zhonghe Zhou, and Zhiheng Li

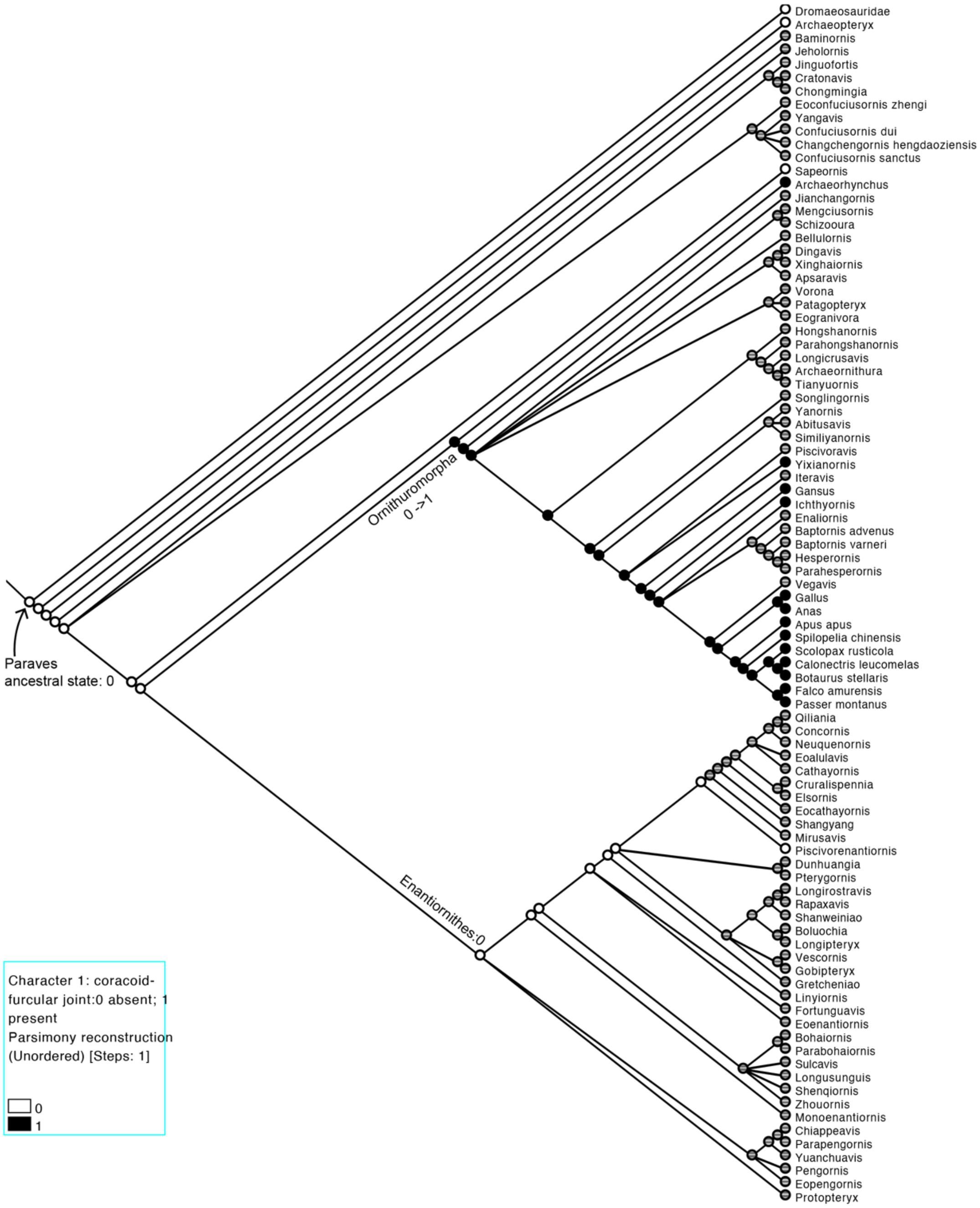

Figure S1. Reconstruction of crocoracoclavicular joint evolution across avian phylogeny. Parsimony-based ancestral state reconstruction shows that a crocoracoclavicular joint evolved once since the most recent common ancestor of ornithuromorphs, and enantiornithines lack the crocoracoclavicular joint as in theropods.

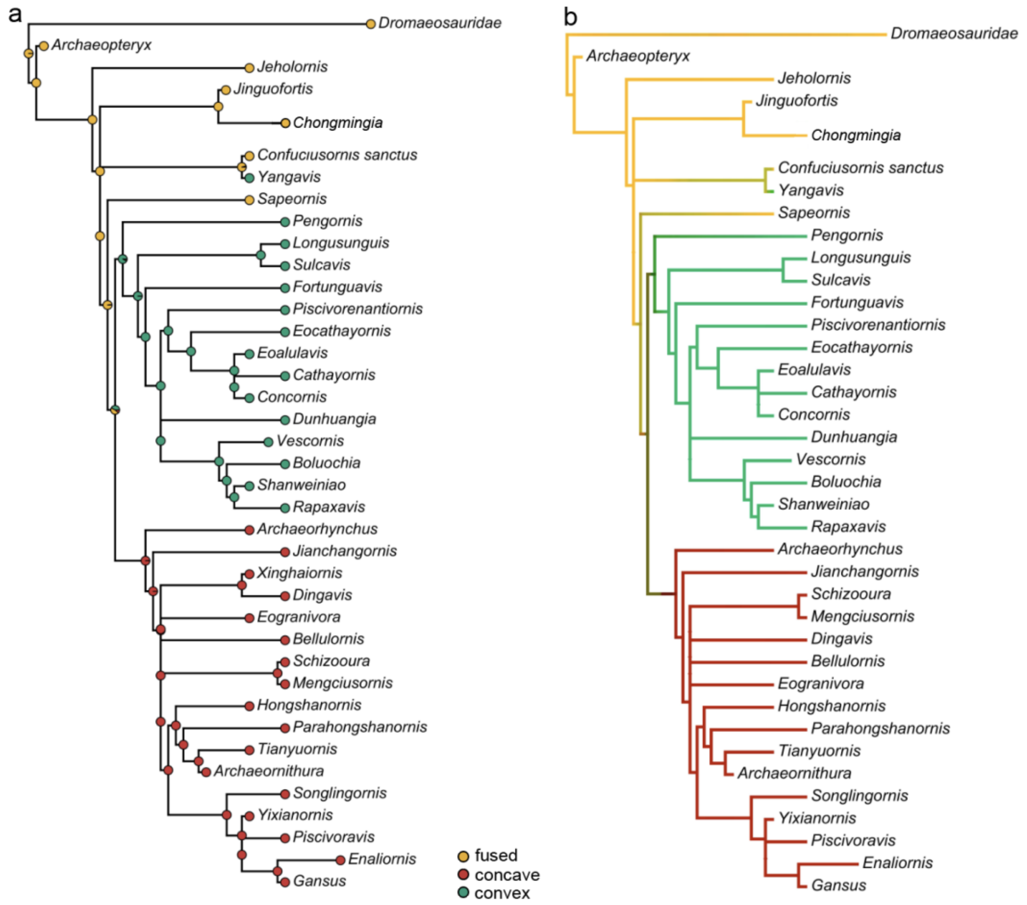

Figure S2. Posterior probabilities (a) and density map (b) from stochastic mapping of ancestor state reconstruction of the morphology of the scapular articulation surface of coracoid among Jurassic and early Mesozoic avians.

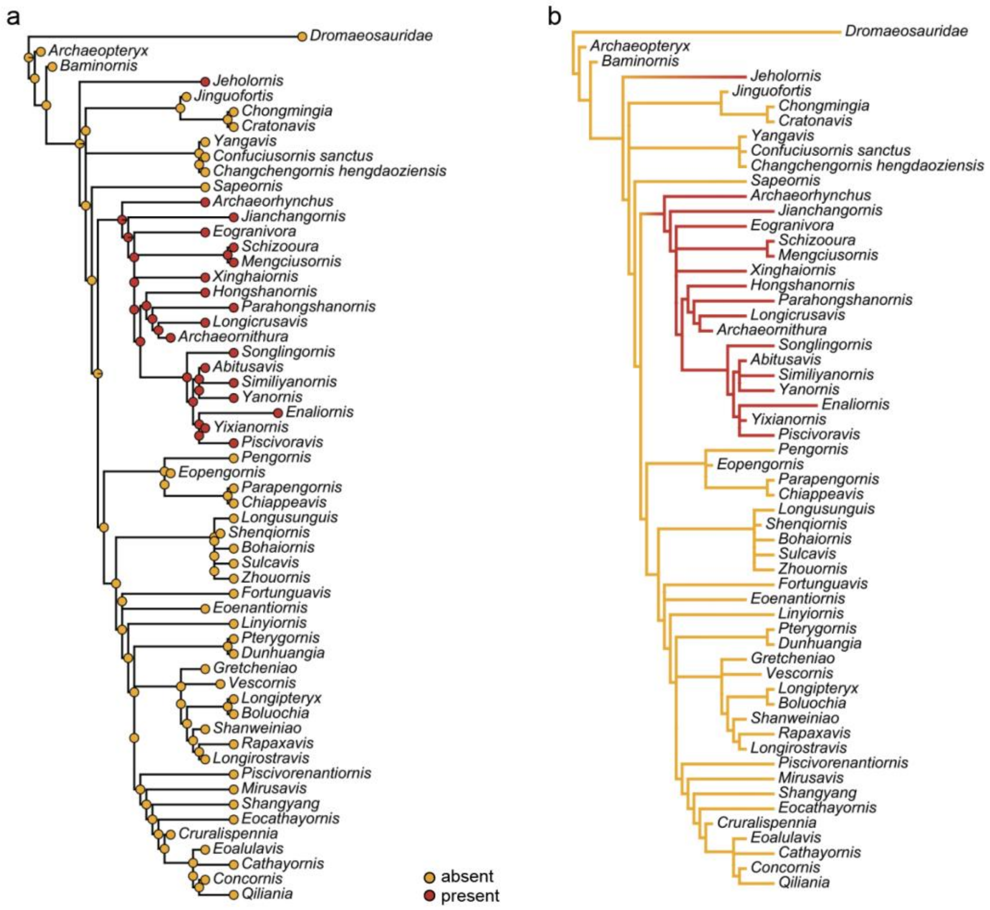

Figure S3. Posterior probabilities (a) and density map (b) from stochastic mapping of ancestor state reconstruction of procoracoid process among Jurassic and early Mesozoic avians.

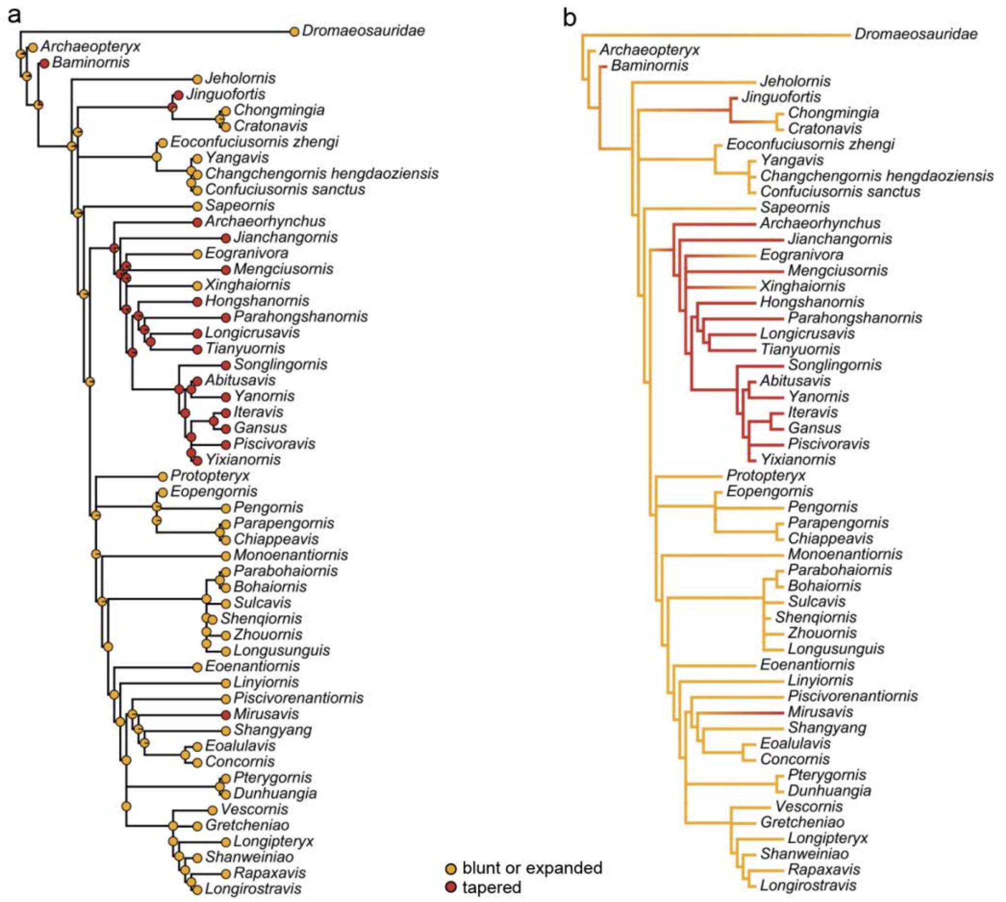

Figure S4. Posterior probabilities (a) and density map (b) from stochastic mapping of ancestor state reconstruction of morphology of the furcular omal ends (epicleidial process) among Jurassic and early Mesozoic avians.

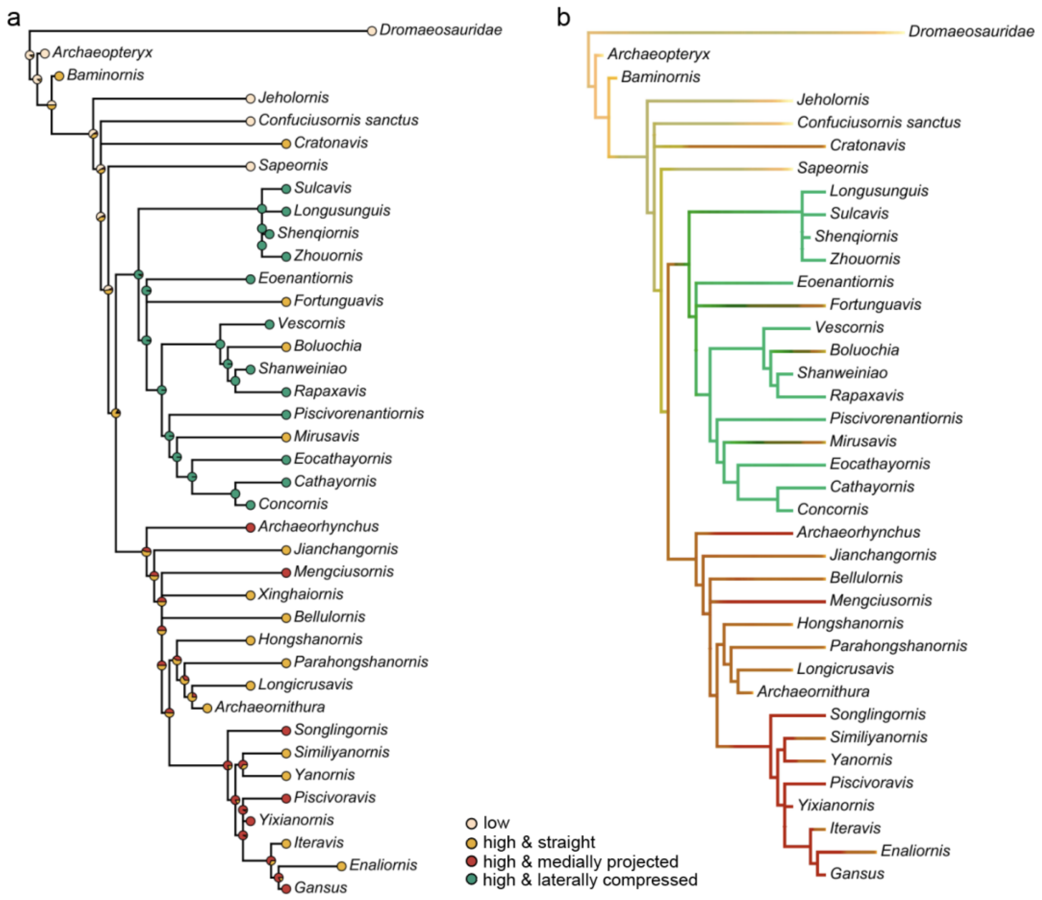

Figure S5. Posterior probabilities (a) and density map (b) from stochastic mapping of ancestor state reconstruction of acrocoracoid process morphology among Jurassic and early Mesozoic avians.

Methods S1. Detailed protocol for ground section, paraffin section and Masson's trichrome staining

The shoulder end of the pectoral girdle of IVPP V 12628 was sectioned for histological analysis. The fragment of the shoulder girdle was embedded in EXAKT Technovit 7200 resin, cured for 12 hours, cut into slices, and polished until the desired optical contrast was reached (slice thickness ~70 μm). Ground section slices were left uncoated, and then observed under natural light using a Nikon Eclipse LV100NPOL and photographed with a DS-Fi3 camera using NIS-Element v4.60 software.

The shoulder end of the extant specimen (*Botaurus stellaris*) was dissected and fixed in neutral buffered formalin, then demineralized in hydrochloric acid and ethylene diamine tetraacetic acid (JYBL-II, Cat DD0017, Leogane) for 48 hours. it doesn't have a IVPP number because it was destroyed during dissecting. After demineralization, the samples were trimmed to proper size and then processed following a standardized protocol for paraffin sections (e.g., see [1]). They were dehydrated with a graded series of ethanol (in 70%, 80%, 90%, 95%, and 100% EtOH for 1 hour each, followed by two additional rounds of 100% ethanol of 1 hour), cleared in xylene (three changes of 30 min each), infiltrated in melted paraffin wax (three changes of 30 min), and embedded manually (Paraplast Plus EMS Cat#19216). The embedded samples were sectioned at 5 μm on a rotary microtome (Leica Biosystems RM2265) and mounted on charged slides (Superfrost Plus, Fisher Scientific).

The sections then underwent a modified Masson's trichrome (MT) stain [2]. Dried slides were deparaffinized in xylene (two changes for 5 min each), dehydrated with a graded series of ethanol (100%, 95%, 90%, 80%, 70% and 50% for 1 min each), rinsed in deionized water for 2 min, stained for 10 min with Mayer's acid hematoxylin (Ruitaibio), rinsed in deionized water for 1 min, stained with Xylidine Ponceau/Acid Fuschin for 2 min (equal volumes of 0.5% xylidine ponceau 2R CI no. 16150 in 1% acetic acid and 0.5% Acid Fuchsin CI no. 42685 in 1% acetic acid), rinsed for 1 min in deionized water, stained for 4 min with 1% phosphomolybdic acid, rinsed for 1 min in deionized water, stained with light green for 90 seconds (2% light green in 2% citric acid, diluted 1 : 10 with deionized water prior to use), and rinsed in deionized water for 1 min. Sections were then dipped twice in 100% ethanol for 10 seconds, cleared in xylene for 4 min (1 min×4), and coverslipped with Permount. The slices were observed under natural light using the same equipment as the ground section slices of the fossil samples.

Methods S2 Identification of the joint type of coracoscapular joint in non-avian dinosaurs and basal birds.

Fused scapulocoracoid are found in ornithischian, as in *Ankylosaur* [3], *Heterodontosaurus* [4], *Changmiania* [5] and *Psittacosaurus*. Especially, the situation in *Psittacosaurus* clearly support that the scapula and coracoid are fused in adult specimens [6]. Among sauropods, fused scapulocoracoid are found as in *Camarasaurus*, *Diplodocus* and *Apatosaurus* [7]. Fused scapulocoracoid are also found in early-derived theropods, as in *Herrerasaurus* [8], *Liliensternus*, *Syntarsus*, *Carnotaurus* [9], *Deltadromeus, Masiakasaurus* [10], and *Raptorex* [11].

In late-derived non-avian theropods, the scapula and coracoid are fused in adult specimens (Fig. 3) [12], as exemplified by *Pneumatoraptor* [13], *Velociraptor* [14], and *Microraptor* [15]. Similarly, in basal birds, a fused scapulocoracoid has been reported in the Maxberg, Solnhofen, and Munich specimens of *Archaeopteryx* [16], Confuciusornithidae [17], and Jinguofortisidae (*Jinguofortis* and *Chongmingia*) [18,19]. While many specimens of *Jeholornis* and *Sapeornis* exhibit separate scapulae and coracoids, such as *Jeholornis* IVPP V 13274 (humerus length 110 mm) [20] and *Sapeornis* IVPP V 13276 (humerus length 123.2 mm) [21], in more ontogenetically mature specimens (e.g., *Jeholornis* STM2-19, humerus length 120 mm, *Sapeornis* holotype IVPP V 12698, humerus length 128 mm) [21] [22] a tightly connected scapula and coracoid is observed. In these taxa the scapulocoracoid are typically preserved together forming an L-shape in lateral view as in non-avian theropods, contrasting with the condition in ornithothoracines in which the two elements are preserved separately [12,21,22]. This indicates the scapula and coracoid of basal birds are connected by cartilage (synchondrosis) during early ontogeny and fuse in adulthood, as in non-avian theropods.

Supplementary References

1. Bailleul, A.M., Witmer, L.M., and Holliday, C.M. (2017). Cranial joint histology in the mallard duck (*Anas platyrhynchos*): new insights on avian cranial kinesis. J. Anat. *230*, 444–460. https://doi.org/10.1111/joa.12562.

2. Witten, P.E., and Hall, B.K. (2003). Seasonal changes in the lower jaw skeleton in male Atlantic salmon (Salmo salar L.): remodelling and regression of the kype after spawning. Journal of Anatomy *203*, 435–450. https://doi.org/10.1046/j.1469-7580.2003.00239.x.

3. Carpenter, K., Kirkland, J.I., Burge, D., and Bird, J. (1999). *Ankylosaurs* (Dinosauria: Ornithischia) of the Cedar Mountain Formation, Utah, and their stratigraphic distribution. In Vertebrate Paleontology in Utah Utah Geological Survey Miscellaneous Publication., D. D. Gillette, ed. (Utah Geological Survey), pp. 243–251.

4. Radermacher, V.J., Fernandez, V., Schachner, E.R., Butler, R.J., Bordy, E.M., Naylor Hudgins, M., De Klerk, W.J., Chapelle, K.E., and Choiniere, J.N. (2021). A new *Heterodontosaurus* specimen elucidates the unique ventilatory macroevolution of ornithischian dinosaurs. eLife *10*, e66036. https://doi.org/10.7554/eLife.66036.

5. Yang, Y., Wu, W., Dieudonné, P.-E., and Godefroit, P. (2020). A new basal ornithopod dinosaur from the Lower Cretaceous of China. PeerJ *8*, e9832. https://doi.org/10.7717/peerj.9832.

6. Hedrick, B.P., Chunling, G., Omar, G.I., Fengjiao, Z., Caizhi, S., and Dodson, P. (2014). The osteology and taphonomy of a *Psittacosaurus* bonebed assemblage of the Yixian Formation (Lower Cretaceous), Liaoning, China. Cretaceous Research *51*, 321–340. https://doi.org/10.1016/j.cretres.2014.06.015.

7. Schwarz, D., Frey, E., and Meyer, C.A. (2007). Novel reconstruction of the orientation of the pectoral girdle in sauropods. The Anatomical Record *290*, 32–47. https://doi.org/10.1002/ar.20405.

8. Sereno, P.C. (1994). The pectoral girdle and forelimb of the basal theropod *Herrerasaurus IschigualastensisI*. Journal of Vertebrate Paleontology *13*, 425–450. https://doi.org/10.1080/02724634.1994.10011524.

9. Weishampel, D.B., Dodson, P., and Osmólska, H. eds. (2004). The Dinosauria 2nd ed. (University of California Press).

10. Carrano, M.T., and Sampson, S.D. (2008). The Phylogeny of Ceratosauria (Dinosauria: Theropoda). Journal of Systematic Palaeontology *6*, 183–236. https://doi.org/10.1017/S1477201907002246.

11. Sereno, P.C., Tan, L., Brusatte, S.L., Kriegstein, H.J., Zhao, X., and Cloward, K. (2009). Tyrannosaurid Skeletal Design First Evolved at Small Body Size. Science *326*, 418–422. https://doi.org/10.1126/science.1177428.

12. Wu, Q., O'Connor, J.K., Wang, S., and Zhou, Z. (2024). Transformation of the pectoral girdle in pennaraptorans: critical steps in the formation of the modern avian shoulder joint. PeerJ *12*, e16960. https://doi.org/10.7717/peerj.16960.

13. Ősi, A., Apesteguía, S., and Kowalewski, M. (2010). Non-avian theropod dinosaurs from the early Late Cretaceous of central Europe. Cretaceous Research *31*, 304–320. https://doi.org/10.1016/j.cretres.2010.01.001.

14. Norell, M.A., and Makovicky, P.J. (1999). Important features of the dromaeosaurid skeleton II: information from newly collected specimens of *Velociraptor mongoliensis*. American Museum Novitates *3282*, 1–45.

15. Xu, X., Zhou, Z., Wang, X., Kuang, X., Zhang, F., and Du, X. (2003). Four-winged dinosaurs from China. Nature *421*, 335–340. https://doi.org/10.1038/nature01342.

16. Wellnhofer, P. (2009). *Archaeopteryx*: The Icon of Evolution (Verlag Dr. Friedrich Pfeil).

17. Wang, M., O'Connor, J.K., and Zhou, Z. (2019). A taxonomical revision of the Confuciusornithiformes (Aves: Pygostylia). Vertebrata PalAsiatica *57*, 1–37. https://doi.org/10.19615/j.cnki.1000-3118.180530.

18. Wang, M., Wang, X., Wang, Y., and Zhou, Z. (2016). A new basal bird from China with implications for morphological diversity in early birds. Scientific Reports *6*, 1–12. https://doi.org/10.1038/srep19700.

19. Wang, M., Stidham, T.A., and Zhou, Z. (2018). A new clade of basal Early Cretaceous pygostylian birds and developmental plasticity of the avian shoulder girdle. Proceedings of the National Academy of Sciences U.S.A. *115*, 10708–10713. https://doi.org/10.1073/pnas.1812176115.

20. Zhou, Z., and Zhang, F. (2002). A long-tailed, seed-eating bird from the Early Cretaceous of China. Nature *418*, 405–409. https://doi.org/10.1038/nature00930.

21. Zhou, Z., and Zhang, F. (2003). Anatomy of the primitive bird *Sapeornis chaoyangensis* from the Early Cretaceous of Liaoning, China. Canadian Journal of Earth Sciences *40*, 731–747. https://doi.org/10.1139/e03-011.

22. O'Connor, J., Wang, X., Sullivan, C., Wang, Y., Zheng, X., Hu, H., Zhang, X., and Zhou, Z. (2018). First report of gastroliths in the Early Cretaceous basal bird *Jeholornis*. Cretaceous Research *84*, 200–208. https://doi.org/10.1016/j.cretres.2017.10.031.
